# Public health emergency preparedness: a framework to promote resilience

**DOI:** 10.1186/s12889-018-6250-7

**Published:** 2018-12-05

**Authors:** Yasmin Khan, Tracey O’Sullivan, Adalsteinn Brown, Shannon Tracey, Jennifer Gibson, Mélissa Généreux, Bonnie Henry, Brian Schwartz

**Affiliations:** 1 Public Health Ontario, 480 University Avenue, Suite 300, Toronto, ON M5G 1V2 Canada; 2 Department of Medicine, University of Toronto, 200 Elizabeth Street, Toronto, ON M5G 2C4 Canada; 3 University Health Network, 200 Elizabeth Street, Toronto, ON M5G 2C4 Canada; 4 University of Ottawa, 25 University Pvt, Ottawa, ON K1N 6N5 Canada; 5 Dalla Lana School of Public Health, University of Toronto, Health Sciences Building, 155 College Street, 6th Floor, Toronto, ON M5T 3M7 Canada; 6 Joint Centre for Bioethics, University of Toronto, 155 College Street, Suite 754, Toronto, ON M5T 1P8 Canada; 7 Université de Sherbrooke, 3001 12 Ave N, Sherbrooke, QC J1H 5N4 Canada; 8 Centre intégré universitaire de santé et de services sociaux de l’Estrie -Centre hospitalier universitaire de Sherbrooke, 300, rue King Est, Sherbrooke, QC J1G 1B1 Canada; 9 Office of the Provincial Health Officer, Ministry of Health, PO Box 9648, Victoria, BC V8W 9P4 Canada

**Keywords:** Public health, Emergency preparedness, Emergency management, Disasters, Disaster risk reduction, Resilience, Qualitative research, Complexity theory

## Abstract

**Background:**

Emergencies and disasters impact population health. Despite the importance of upstream readiness, a persistent challenge for public health practitioners is defining what it means to be prepared. There is a knowledge gap in that existing frameworks lack consideration for complexity relevant to health systems and the emergency context. The objective of this study is to describe the essential elements of a resilient public health system and how the elements interact as a complex adaptive system.

**Methods:**

This study used a qualitative design employing the Structured Interview Matrix facilitation technique in six focus groups across Canada. Focus group participants were practitioners from public health and related sectors. Data collection generated qualitative data on the essential elements, and interactions between elements, for a resilient public health system. Data analysis employed qualitative content analysis and the lens of complexity theory to account for the complex nature of public health emergency preparedness (PHEP). The unit of study was the local/regional public health agency. Ethics and values were considered in the development of the framework.

**Results:**

A total of 130 participants attended the six focus groups. Urban, urban-rural and rural regions from across Canada participated and focus group size ranged from 15 to 33 across the six sites. Eleven elements emerged from the data; these included one cross-cutting element (Governance and leadership) and 10 distinct but interlinked elements. The essential elements define a conceptual framework for PHEP. The framework was refined to ensure practice and policy relevance for local/regional public health agencies; the framework has ethics and values at its core.

**Conclusions:**

This framework describes the complexity of the system yet moves beyond description to use tenets of complexity to support building resilience. This applied public health framework for local/regional public health agencies is empirically-derived and theoretically-informed and represents a complex adaptive systems approach to upstream readiness for PHEP.

**Electronic supplementary material:**

The online version of this article (10.1186/s12889-018-6250-7) contains supplementary material, which is available to authorized users.

## Background

Emergencies and disasters impact population health, as we face diverse hazards influenced by complexities in our environment, demographics and social constructs. Novel and re-emerging infectious diseases continue to cause morbidity and mortality and can rapidly spread beyond borders. In Canada, wildfires have resulted in large population evacuations, air pollution and deaths [1–3]; floods are an annual risk causing displacement of Indigenous communities, urban infrastructure damage and adverse health impacts [4–6]. The 2013 Lac-Mégantic train derailment and explosion resulted in 47 deaths, environmental contamination, and adverse mental health impacts [7, 8]. Reducing risks and the short and long-term impacts of all-hazards emergencies on population health is a key responsibility for the public health sector [9, 10]. Public health plays a critical role in working with health and non-health sectors responsible for preparing for and responding to emergencies, yet have limited resources and competing priorities in delivering community health protection and promotion programs. While emergencies tend to raise awareness about the significance of being prepared, public health agency readiness activities operate largely in the background until an event occurs. Despite the importance of upstream readiness, a persistent challenge for public health practitioners is defining what it means to be prepared [11–14].

Defining preparedness using an evidence-informed approach is challenging, due to the general lack of evidence to inform disaster risk reduction (DRR) for public health [15]. To our knowledge, there are few published frameworks for public health emergency preparedness (PHEP) or DRR which used empirical methods in derivation [11, 16–20]. Some frameworks reflect authors’ opinion [11], others describe some form of stakeholder consultation process; however, the methodology used to achieve consensus lacks detail [17, 21] and there is no widely accepted framework that can be used to guide and compare efforts.

In reviewing the extant literature, we note most country-specific PHEP frameworks were developed in the United States (US) and have unclear relevance to other settings with substantially different health systems and governance structures [16, 22, 23]. Outside the US, the European Centres for Disease Control has adapted a US model [19, 24] in considering core competencies for cross-border threats across the European Union [20, 25]. Globally, there are a number of frameworks and initiatives that have relevance to PHEP and DRR [26–28]. The World Health Organization (WHO) framework to inform emergency preparedness is based on consultation with global stakeholders and lessons learned [28]. The framework is designed to be relevant to health systems globally and emphasizes national, subnational and local connections. The United Nations Sendai Framework for DRR has four key priorities and takes a whole-of-society approach [27]. It expands on its predecessor, the Hyogo Framework, with specific reference to the health impacts of disasters and reducing risks. These frameworks highlight the importance of national action and global collaboration to improve health system preparedness and reduce disaster risks; however, empirically-derived and contextually-relevant evidence to inform public health actions for local/regional public health agencies remains a knowledge gap.

In the aftermath of the 2014–16 West Africa Ebola Virus Disease outbreak, the WHO called on all countries “to create resilient integrated systems that can be responsive and proactive to any future threat” [29, 30]. Resilient systems have been defined as: “those that rapidly acquire information about their environments, quickly adapt their behaviors and structures to changing circumstances, communicate easily and thoroughly with others, and broadly mobilize networks of expertise and material support” [31]. A challenge with existing PHEP frameworks is an inconsistency of elements or components, and lack of consideration for PHEP as part of a system [22, 24]. For example, social capital was missed in one framework, when disaster resilience research has noted the importance of both hard, or physical, infrastructure and soft, or social, infrastructure [32, 33]. Further, none of the identified frameworks articulate a consideration for the role and contribution of values in PHEP, yet Canadian experiences have underscored the importance of ethics and values. Questions such as “Who will get the limited supply of antivirals and vaccines?” cannot be informed solely by science, and failure to acknowledge underlying value judgments can result in a loss of public trust and low health worker morale, impacting community recovery after an event [34–37]. Incorporating ethics and values explicitly could add to the legitimacy and usefulness of a PHEP framework.

The knowledge gap that exists with PHEP frameworks thus extends to their comprehensiveness to reflect the dynamic and social context of public health emergencies and the complex public health system. Resilience-oriented interventions for community disaster preparedness have been proposed by accounting for the complexity of the emergency context and we argue that complexity is the backdrop that must guide strategy in re-framing PHEP [32]. Complexity as a theoretical approach is described as a set of concepts and analytic tools that can be applied to understand various properties of systems and is potentially useful in developing management or intervention strategies [38–44]. This theoretical foundation is necessary in developing a PHEP framework to provide the depth required to account for the emergency context, a complex adaptive system like the public health system, and building resilience.

In this paper we present an empirically-derived and theoretically-informed framework for emergency preparedness to inform local/regional public health agency practice. Our objective is to describe the essential elements of a resilient public health system and how the elements interact as a complex adaptive system.

## Methods

This study is part of a two-phase project that aims to advance performance measurement for public health emergency preparedness (Additional file 1) [45]. The overall project approach is an exploratory, sequential, mixed methods model to inform the development of indicators for PHEP (Additional file 2). The two-phase project is based in Canada and this paper reports findings from phase 1, corresponding with the first objective stated above. Several papers are planned from the overarching project.

For phase 1 we used a qualitative design employing the Structured Interview Matrix (SIM) facilitation technique in six focus groups across Canada [46, 47]. As an applied public health project, the study was structured to include Integrated Knowledge Translation (iKT) and knowledge users who are defined as individuals likely to use research results to make informed decisions about health policies, programs and/or practices [48]. A steering committee of knowledge users provided input on study milestones to ensure that the research was relevant and useful to the field [48]. Detailed reporting for this qualitative study is found in the Consolidated Criteria for Reporting Qualitative Research checklist (Additional file 3).

In planning the SIM focus group sites, we included anglophone and francophone communities and focused on representation of Canadian regions with diverse experiences with public health emergencies. Recent emergencies and disasters include emerging infectious disease outbreaks, industrial disasters, wildfires, extreme weather and planned mass gatherings. The six sites spanned four provinces in Canada, representing Atlantic, Central, and Western regions. The focus groups were held in two urban centres, three urban-rural communities, and one rural location [49–51]. Ethics approval was obtained from the Public Health Ontario and University of Ottawa Ethics Review Boards.

We used purposive and snowball sampling to ensure diversity of expertise and involvement of people in senior decision-making roles [52, 53]. Between eight and 40 participants can participate in one SIM session [46]. Optimal rich data generation balanced with feasibility of recruitment and flow of the session is observed with 16–24 participants, thus informing our target SIM size. Participants represented the public health system at multiple levels (local/regional, provincial, federal), with an emphasis on local/regional public health as the unit of public health delivery in Canada. Participants from other sectors involved in aspects of PHEP were recruited to reflect the complex adaptive system. Public health participants consisted of decision-makers or experts at multiple levels. Participants from health care and the health system consisted of senior decision-makers or professionals with expert knowledge of emergency preparedness for the health care sector (e.g., primary care, acute care) and linkages with public health. Government and policy-maker participants included decision-makers from health ministries or emergency management agencies of government, with expert knowledge of emergency preparedness. Community, social service or private industry participants included senior decision-makers or professionals in community organizations with expert knowledge of and roles in emergency preparedness and service provision for high-risk populations.

For recruitment, the research team and knowledge users generated a list of potential participants and organizations. Invitations were distributed by email. Informed consent was obtained from each research participant prior to participation in the study. Data generation occurred over a three month period from April to June 2016. Focus groups were held in a professional meeting space during working hours separate from participants’ workplaces and were 2.5 h in duration.

The three-part SIM facilitation technique consists of one-on-one interviews, small group and plenary discussions (Additional file 4) [46]. Data collection for the SIMs was anchored around four questions developed by the research team, refined with knowledge user input, and piloted. Equity and ethical considerations related to emergencies informed the data collection approach. The final four questions are found in Table 1. The English focus groups were facilitated by a study co-investigator (TO) who is a doctoral-trained qualitative researcher with extensive experience in implementing SIM focus groups. The French focus group was facilitated by an experienced bilingual facilitator who was involved in developing the SIM technique for application in research settings. Focus group resources were translated to French by a professional translator. Both facilitators have experience collaborating on SIM implementation for research and use a consistent approach. Facilitators’ credentials and experience in the field of emergency/disaster research were shared with participants.

**Table 1:** Structured Interview Matrix focus group questions

	English version	Version française (French version)
1.	What are key elements of preparedness for the public health system pertaining to outbreaks and infectious disease emergencies?	Quels sont les principaux éléments de la préparation du système de santé publique concernant les urgences liées aux éclosions et aux maladies infectieuses?
2.	What are key elements of preparedness for the public health system pertaining to natural disasters or anthropogenic emergencies?	Quels sont les principaux éléments de la préparation du système de santé publique concernant les urgences liées aux désastres naturels ou les urgences anthropiques?
3.	What makes the public health system resilient?	Qu’est. qui fait que le système de santé publique est. résilient?
4.	Based on your emergency preparedness or response experiences, what situations have you encountered where you have had to consider values or fairness?	Selon votre degré de préparation aux situations d’urgence ou de vos expériences en matière d’intervention, dans quelles situations avez-vous dû tenir compte des valeurs ou de l’équité?

Each focus group was attended by the facilitator, the Principal Investigator and two research team members in addition to research participants. The facilitators interacted directly with participants for data collection. For the majority of sites, participants had no knowledge of any research team members prior to the SIMs. Exceptions to this were knowledge users from the steering committee who participated in SIMs in their region. A small group of participants at one focus group were familiar with the facilitator (TO) from prior research initiatives.

The data generated during the SIM sessions included field notes, audio-recordings of small and large group discussions, and observations from the research team [46]. Participant checking occurs in the third part of the SIM focus group, as participants confirm the data during the plenary discussion. Audio-recordings were transcribed verbatim at the Resilience and High Risk Populations Research Lab at the University of Ottawa. Transcriptions were performed by students supervised by a graduate trainee in qualitative methods and were all familiar with the SIM method and process. Each transcript was checked for quality and accuracy. The data from the French focus group were translated to English for analysis after transcription.

Qualitative content analysis was conducted in several steps (Additional file 4) [46, 54]. Interview field notes were used to develop the coding grid by four team members. The final coding grid is provided in Additional file 5. Transcripts from small and large group deliberations were coded by two research team members iteratively until agreement was reached on application of the grid to the data. Subsequently, one team member coded the remaining transcripts. NVivo™ 10 software was used for qualitative data management. The coding reports were analyzed inductively to identify emergent themes, which were revised until consensus was reached.

The themes represent collective responses at the system level for all the questions pertaining to the essential elements of PHEP, resilience in the public health system, and the consideration of ethics in PHEP. The research team involved in analysis included both insider and outsider perspectives in terms of positionality in relation to the PHEP field. Expertise of the team included public health, health emergency management, disaster risk reduction, ethics, and health systems and services. The themes were developed iteratively and went through several revisions incorporating input from the knowledge user steering committee.

Complexity theory was applied as a lens to the themes by two authors (TO and YK) to explain how different elements of the framework account for tenets of complexity. Theory on complex adaptive systems, health systems and emergency management were used to inform our complexity approach [40, 42]. The seven tenets applied in analysis relate to characteristics of complex systems (dynamic context; interconnectivity; feedback loops; emergence) and change (adaptability; self-organization; non-linearity) [40]. Each thematic description below finishes with an explanation of its complexity as an element of PHEP. A more detailed description of the methods and results from the complexity analysis will be presented in a separate paper.

## Results

A total of 262 individuals were approached to participate in a SIM at a fixed date, time and place in their region. We over-sampled given travel required away from participants’ workplaces and the substantial time commitment during working hours. The number of participants across Canada who accepted the invitation was 146. In some instances interest exceeded the capacity of the focus groups. In deciding allocation for participation, priority was given to ensure a diverse participant mix based on the sampling strategy. Of the 146, 19 individuals across the six sites were unable to attend the focus groups. Reasons cited included professional conflicts and illness. Three individuals who did not attend sent a delegate.

The final sample represents a total of 130 participants who attended the six focus groups. Participation in the focus groups ranged from 15 to 33 across the six sites. The smaller sizes for focus groups corresponded with smaller sized communities. Focus group size for each site in relation to geographic regions in Canada is displayed in Table 2. Focus group sites are denoted by an anonymized letter. Diversity in the sample was achieved with representation across the complex adaptive system of PHEP is also displayed as Table 2.

**Table 2:** Characteristics of Structured Interview Matrix focus group sites

Site	Geography	Urban vs rural characteristics	Public health (all levels), *n* = 44	Health system and health care, *n* = 31	Government and policy-makers, *n* = 41	Community, social services, and/or private industry, *n* = 14	Total, *N* = 130
A	Atlantic	Rural	3	3	7	2	15
B	Western	Urban-rural	9	4	2	0	15
C	Central	Urban-rural	6	4	4	4	18
D	Western	Urban	9	4	7	4	24
E	Atlantic	Urban-rural	4	6	13	2	25
F	Central	Urban	13	10	8	2	33

The results of data analysis are presented in two sections. The first presents the emergent themes resulting from qualitative data analysis. The themes represent the essential elements of PHEP. For the remainder of this paper the themes will be discussed and referred to as elements. Eleven elements emerged from the data; these included one cross-cutting element (*governance and leadership*) and 10 distinct but linked elements. The second section presents the developed framework and depicts the relationships of each element to the role of public health in the complex system of emergency management.

### Governance and leadership: Integrated structures, partnerships and accountabilities with clear leadership to support a coordinated, interoperable system

Leadership as a concept is related to, but separate from governance. Both are foundational, cross-cutting elements for PHEP and form the basis for the PHEP system. The integration of public health with health and non-health sectors was identified as essential. Leadership is a mechanism for articulating the role of public health agencies in responding to emergencies from different types of hazards, and ensuring alignment between governance and agency plans.

*“…before the plan can be developed you need to create a mandate. First clearly define the role of public health, then develop a plan to support that role…we know what our role is – other people may not understand.” (Site F)*

Coordination was highlighted as an important output of an integrated system of emergency management, and a lack of integration was described as a precursor for inconsistency and uncoordinated emergency actions. Finding the right balance of separation and connectedness in governance is paramount; where organizations are specialized yet still coordinated within an integrated system. Views on whether there is a need to bridge or to dismantle silos in public health varied across the sites, as evidenced in the following quotations:

*“You don’t want to pull down your silos, because those are your pillars of excellence, you need to build proper bridges in between those silos.” (Site A)*

*“It’s breaking those silos down. But I think we need to do it long before an incident occurs. So I think preparedness is the perfect time to begin building those relationships. And looking for those champions in each area… finding your experts, finding your resources.” (Site B)*

When we applied complexity theory to *governance and leadership*, the tenet of interconnectivity stood out as an important consideration when examining the role and influence of silos, and adaptively bridging or dismantling as required. Pre, during or post-disaster different sectors, organizations, and jurisdictions must collaborate to adapt to changing situational awareness. Governance structures which are sensitive to interconnectivity will support innovation when flexibility is required.

Clarity – in relation to authority, roles and responsibilities – was emphasized across all sites. The importance of identifying a lead agency and authority was underscored in relation to *governance and leadership*. Understanding where public health fits in the governance structure is important, as well as the governance and management models agencies use to organize their internal structure and its interface with the system. Incident Management or Command Systems were discussed in relation to the characteristics they enabled, such as adaptability. System flexibility is essential in a disaster, particularly in terms of interoperability. As a disaster unfolds, the context influences the way an emergency plan can be implemented, within and across organizations.

*“For infectious disease it’s fairly clear who has that lead … public health is the lead in that. But when it comes to other kinds of disasters it’s not as clear where does public health actually fit within all of that structure. So, we need to establish that leadership and whoever is going to take that leadership needs to know where does everybody fit.” (Site F)*

The role of public health as a collaborator was emphasized in discussions on leadership. In addition, roles related to prioritizing preparedness for emergencies, and leadership influencing a culture of preparedness within public health agencies. Leadership is dynamic and can emerge as organizations connect and understand the different and common constraints they face. Leaders with skills to bring different people and organizations to the planning table emerge as the planning and/or response evolves. The need for innovative or improved governance structures may also emerge as leaders assume new roles in planning and preparedness and the different factors influencing collaborative action are discussed.

### Planning process: Develop a plan through a dynamic, collaborative planning process

This theme underscores the value of the process of planning in public health preparedness. Planning is important for clarifying roles and responsibilities, and understanding organizational structures and functions. Equally important is the development of relationships, which contributes to efficiency in preparedness and response. As evidenced in the following quotation, participants de-emphasized the static nature of the plan as a “book” or document, and discussed the *planning process* as the anchor for ensuring system adaptability and responsiveness.

*“We call it planning, but it is more developing a process. … in the sense of the [health system structure] where it’s scalable and it encompasses all portions of it, not just public health. So don’t think of it just as a plan like a book sitting on a table, it’s more the whole aspect of everything that encompasses the response process.” (Site B)*

The *planning process* links with other elements of PHEP, as described by this participant and developed further in this section.

*“And in the process of developing the plan, collaborating or communicating with others in the development of that plan. So, making sure that we are engaging each other, and stakeholder communities, in that plan so that we can develop role clarity as part of that process and get to know each other. Identifying the tools and resources that we would need to respond would be part of planning, and then investing in the training and, exercising that plan.” (Site A)*

PHEP planning is a complex process, given the multiple influences and interdependencies in public health emergencies. Planning must consider changing population demographics, political and environmental factors. It must take into account not only local and regional contexts, but also global influences. When changes occur within a complex adaptive system, interdependencies create a ripple effect and impact other parts of the system. This creates the opportunity for a feedback loop, which provides information that can be used to adapt. Readiness for emergencies depends on the ability of a system to adapt to changing circumstances, thus planning must be updated on an ongoing basis.

### Collaborative networks: Develop relationships, partnerships and strong networks

Linked with *planning process*, collaboration emerged as a strong concept in participant discussions on resilience, particularly with respect to efficiency in response activities and organizational learning. *Collaborative networks* can support readiness, response and recovery across multiple levels of the system, and include stakeholders outside the public health system, whether in clinical care, emergency management, government or the private sector.

*“Stakeholder engagement, so again the importance of this connectivity across the system and with others outside of the health system.” (Site D)*

*“Well, how do you make a public health system resilient is collaboration and that’s straightforward. Many groups working together, being able to understand what each other’s roles are and what their strengths and weaknesses they bring; it’s that collaborative framework that’s going to make your public health system resilient.” (Site E)*

*Collaborative networks* are essential for accessing needed expertise, which in turn contributes to awareness and adaptive management as new knowledge is created and context changes. Sharing of expertise can be formal or informal, but is the cornerstone of strategic renewal in organizational learning. The concept of the networked system bringing together skillsets and resources contributes to emergence in the system. System behaviour can be unpredictable related to interactions between its components, and emergence pertains to how system behaviour emerges and the whole being more than the sum of its parts [40]. Further, networks are often the source of non-linearity, which may be positive or negative in nature. Non-linearity depends on feedback where cause and effect are not proportional [40]. Both positive and negative feedback contribute to change in the system as actors and parts interact over time.

*“Sharing resources and skillsets, because we all bring to the table different skillsets.” (Site C)*

*“It’s important to have those trusted, open relationships to develop our individual skillsets, but also our collective ones.” (Site C)*.

Trust develops through collaboration which can strengthen the connectedness and resilience of the networked system. In relation to complexity, the interconnectivity tenet is integral to the idea of partnerships and strong networks. PHEP is highly dependent on actors and the relationships between actors leading to adaptive response. Networks are inherently dynamic; people or actors within networks change, their relationships and personal networks are dynamic, and their experience / expertise also change. This ever-changing profile contributes to the dynamic nature of the entire system.

### Community engagement: Understand and engage with the community

Collaboration with the community intersects with planning, in that it enables the consideration of community risks, cultural considerations and experiences. Planning that takes an inclusive approach and engages the public promotes common understanding of risks, assets and values, and can facilitate transparency between public health agencies and the community. Participants noted the link between resiliency among the public and the resilience of the public health system.

*“Why Public Health is resilient is because the people are engaged…because of that, the mobilization of the vital elements is easy, and their engagement is built up through past experiences.” (Site C)*

The ability for *community engagement* to build trust between public health agencies/leaders and the public was recognized as crucial to public health protection for emergencies, and important in building long-term community support for emergency preparedness, response and recovery. Transparent and responsive engagement and communication with the public promotes credibility and trust for urgent population health messages such as boil water advisories or evacuation.

*“It’s the public trusting us as agencies… or the agencies being trustworthy enough that when the time comes the public goes ‘I feel like I can rely on this as good pertinent information’.” (Site D)*

*Community engagement* is a mechanism to assist with difficult decision-making; specifically involving community groups in planning decisions that may impact them. Engagement with communities can promote the consideration of assets within particular groups, rather than focusing solely on deficits or vulnerabilities. Connection between public health agencies and the population whose health it aims to protect was described as essential to resilience in the public health system.

*“There was no engagement with the communities that the plans were most meant to affect. So back to your equity question, there was a lot of concern in pandemic preparedness for vulnerable populations. So we brought vulnerable groups together who were identified in the plans and they told us ‘why are you calling us vulnerable? Your plan is actually what makes us vulnerable in the first place’...” (Site F)*

Communities are part of the complex system and interconnectivity is inherent. When considering *community engagement*, it is important to understand where the influential connections are in the community and how this can support or create challenges for response plans. Connections are important assets for public health activities across all phases of a disaster. They represent specific knowledge and communication channels that can support resilience.

It is important to recognize that *community engagement* is dynamic at different times and places, different phases of a disaster, and over time as community members change. *Community engagement* will emerge as the context changes and as opportunities are presented to contribute. Inclusive opportunities for engagement provide feedback loops to develop and contribute to innovation, situational awareness, and mobilization of resources.

### Risk analysis: Robust understanding of community hazards and risks

Understanding risk is essential to inform planning; *risk analysis* is a critical contribution of public health agencies during an adverse event. While assessment of risk is a crucial first step in proactively understanding the dynamic and interconnected context of each community, it is important to follow it up with analysis and strategies to build capacity for resilience.

*“… analytical capacity as a prerequisite to resilience. If the situation isn’t analyzed correctly, you’re going to have responses that are not appropriate. ... Analytical capacity, even being proactive, even before, in the area’s risk profiles, that can make all the difference [for] resilience.” (Site C)*

Pre-existing disparities in the social determinants of health were emphasized as important pieces of the picture in understanding risk across a community and within specific populations. This underscores the link between *risk analysis* and *community engagement* as essential elements of PHEP, and the importance of inclusivity as a principle in planning.

*“Social risk factors… poverty, disability, versus clinical, physical… pre-disaster someone may be a person with a disability or may not be a person with a disability, but when a disaster happens they may become higher-risk. Like, someone with mental illness for example likes routine all set, but when an emergency happens they’ve got to leave their home, they then become a person at risk.” (Site E)*

Conducting a thorough *risk analysis* implies strong partnerships and information-sharing capabilities in accessing information from other sectors. *Risk analysis* is an ongoing process for complex adaptive systems. Context is constantly changing. Situational awareness must be continually updated and shared – to understand the risk profile at any given time. The nature of risk within a complex adaptive system creates a situation where it may be challenging to anticipate consequences. Complex interdependencies can quickly cascade into serious operational issues when risk is realized in one highly interconnected part of the system. *Risk analysis* provides a means to understand environmental and contextual influences, and make contingency plans to account for interdependencies.

### Surveillance and monitoring: Timely information to provide situational awareness and guide action

The essential element of *surveillance and monitoring* incorporates early detection and warning; situational awareness; and formal surveillance systems. *Surveillance and monitoring* includes routine public health surveillance such as formal lab-based and emergency department surveillance, some of which are legislatively mandated. Other information sources such as global situational awareness facilitate “early warning” that enable initial alerts of emerging risks to public health authorities and the broader system.

*“We need to have intelligence to be able to know when to react… Because in case of emergency, you want to actually ensure that if something does happen, then you want to ensure that the right people are told in a timely fashion.” (Site B)*

The essential nature of *surveillance and monitoring* as an element was clear; however, there were apparent discrepancies in how well-functioning and resourced surveillance systems are across Canada, to support management of emergencies and disasters.

*“For monitoring/surveillance, well, the importance of epidemiological monitoring, of what’s happening elsewhere in the world to be able to increase our own vigilance, then adapt the network’s capacities in greater detail based on that.” (Site C)*

*“A big one, particularly around monitoring, gathering, and real-time information that comes back and feeds back to the people that need to know about it.” (Site F)*

Surveillance and situational awareness connect different parts of a complex system, and relate to *collaborative networks*, *risk analysis* and *communication*. For accurate *surveillance and monitoring*, it is important that interconnectivity be considered, to ensure actions taken in one part of the system are assessed in terms of how they may affect another part of the system. When one part of the system becomes aware of information that can impact another part of the system, it is important to share that information; these are essentially feedback loops which update situational awareness and inform decision makers if different actions must be taken.

### Practice and experience: Invest in testing and practicing plans and processes

Whether practice occurs through simulations, exercises or experience in actual events, it was deemed essential for building capacity for response. Practice is a mechanism by which plans can be tested, gaps identified and processes tweaked. Two dimensions of experience emerged: 1) *knowledge*, skills and training of the workforce; and 2) *application* of the skills or training. The latter emphasizes practicing/testing plans and developing experiential learning for staff. Experience enables teams to revise protocols and provide feedback on parts of the plan that are no longer relevant or effective.

*“… training, simulation. That really came up a lot. But not just training on the contents of toxicological evidence and all that, but also training on how to work in emergency mode, with whom, and then everyone’s roles and responsibilities.” (Site C)*

*Practice and experience* enables and reinforces other elements of PHEP. Roles can be clarified, relationships developed and planning processes refined. *Practice and experience* can therefore function as a strategy to build resilience in the system.

*“… roles, and responsibilities, and relationships are really clear and have been developed in advance. Those might be developed in advance through a variety of ways including through the planning process, through training, through exercises and also just through experience, so we get involved through responses together…” (Site D)*

Evaluation of *practice and experience* enables a jurisdiction to understand if it is ready and potentially resilient to a threat. *Practice and experience* thus links with *learning and evaluation* (described below) and it is important that measures are collected consistently and completely. If practice is not possible through experience, it is closely linked with resources. Challenges arise if funding for relevant practice or simulations is lacking.

*“We don’t have any funding for drills or exercises to ensure that we are being resilient, because we have not had these kind of events we really do not really know what the level of our resilience is, it’s theoretical.” (Site D)*

Through a complexity lens, *practice and experience* support resilience through feedback and co-evolution of the actors within the public health system and the entire system as an entity. Actors within the system must have the confidence and skills to adapt their activities and decisions as situational awareness changes. As teams or actors adapt, they co-evolve over time so that both actors and the system evolve together, based on practical experience.

### Resources: Ensure dedicated resource capacity and mobilization capacity

Discussion around resources focused on two large aspects which are represented as distinct elements: first, physical, structural, and financial resources; and second, human and workforce assets. Human resources and workforce assets are discussed as a separate theme below. Structural/physical resources were described in terms of the capacity for systems and infrastructure to support elements of PHEP, such as adequate systems for risk analysis. *Resources* underpins multiple elements that require sufficient infrastructure and investment to function effectively.

*“Resources is another thing, you just, you can’t do this on the cheap. If you’re going to do it, you’ve got to pay for it… Resources to invest in the actual structures and then the resources that are available to sustain function…as separate from people…” (Site D)*

Participants highlighted intangible assets, such as time and organizational support so people actually have time to do emergency preparedness. Creating space in peoples’ workloads for preparedness activities requires not only a cultural shift, but financial investment to show organizational commitment to ensuring *resources* are there for PHEP.

Difficult decisions surround allocation of limited *resources*. It was identified that there is a need for proactive decision-making around resource allocation as part of planning. This includes development of processes to assist with challenging resource decisions that emerge and are time-sensitive during the response phase. Transparency and consideration of diverse values and priorities in the community are also important for building trust.

With respect to the complexity of resource allocation for emergency response, the need for adaptability is paramount. At any time of year and during any phase of an emergency, the asset-profile of a community is dynamic. Feedback loops provide information about available resources that can be mobilized or gaps where resources need to be secured to enhance preparedness. When resources are limited, not available or hard to mobilize, self-organization will occur naturally as people and organizations work innovatively with what they have.

### Workforce capacity: Develop and support knowledgeable, skilled and resilient staff

People and social infrastructure emerged as essential in PHEP. Knowledgeable people developed through training, experience, and possessing specialized expertise, are crucial assets to support resilience. Training was described in terms of content expertise, but also as relevant skills important for emergency management, such as communication and collaboration. Having sufficient human resources within the system provides redundancy (for back up) and supports interoperability.

*“I think what we’ve heard is that people— it’s about proper training and redundancy. And so, you have people that are well trained and there is enough of them. And this is all around being in the public health system being resilient, right? Staff resilience coming from being adequately funded, trained.” (Site D)*

Adaptability across different workforces involved in public health emergency response is a necessary ingredient for resiliency; and is part of the complexity. When unexpected events appear – or the context changes within a current event – the workforce must be prepared and skilled at adapting its response strategies. Supporting staff to deal with challenges during an event is an important organizational role and demonstrates reciprocity [55]. Staff may be directly impacted by an emergency as individuals in the community, or experience distress through their experience as responders. Public health agencies are critically dependent on the resilience of staff; therefore, having some redundancy in the system builds adaptive capacity to support resilience.

### Communication: A strategy to deliver clear, consistent messaging across networks and the public

*Communication* involves multiple audiences and purposes, such as delivering information to promote public action/behaviour change, providing guidance for health care professionals, or sharing information internally with staff. Strategy is an essential part of communications planning and includes determination of the amount of information, the audience, messaging methods and content. *Communication* was described largely as communication out from public health agencies; however, mechanisms for gathering information and feedback also emerged.

*“Communications, knowing who to call… targeting the risk communications, keeping the simple messaging that people can understand, actions they need to take. And it goes both ways. I think there’s the public communications but there’s also the internal for the practitioners” (Site D)*

*Communication* supports adaptability in PHEP; as situational awareness changes, decisions must be made about how to share information with different stakeholders. *Communication* links with the other themes in this framework, as participants clustered it with *resources* and infrastructure as critical to support response capacity.

In participant discussions related to resilience of the public health system, communication became subsumed under discussions on collaboration. Discussion around communication as it relates to a resilient health system was described in terms of public engagement, and how an engaged and informed public can be more resilient. Trust built through community engagement promotes effective communication and public action.

*“Internal and external communications, so communications within your organisation making sure people understand…and then that external communications out with the public.” (Site E)*

*“If we can educate … if we can have a good public communication system so we can get the public to participate in generating resilience. And we’re going to see that it’s often there that being more resilient starts.” (Site C)*

*Communication* as a specific activity represents an essential element of PHEP; however, the networks, relationships, feedback and collaborative processes that enable effective communication are part of the inherent complexity in public health, and are prominent in building resilience. Adaptability and feedback are central to *communication*. Feedback loops provide information about how messages are being received within and outside the system. If communication is not available or accurate, people will explore different channels to obtain information they need to perform their roles or reduce uncertainty. Informal networks provide innovative channels for communication, particularly when channels are down or ineffective.

### Learning and evaluation: Evaluation as a strategy to build resilience

Learning was described by participants as adaptability during emergency response, and in preparing for future events. Learning links with other essential elements for PHEP, such as *surveillance and monitoring*, with a forward thinking lens.

*“I think we learn from our mistakes. And I think the back-end of any good plan is to make sure that when there is an incident that there’s a debrief, robust after-action or post-incident review of what was done correctly, what was done incorrectly, where could we improve and what did we learn from this... Now that helps to inform how we respond going forward.” (Site F)*

Developing a *learning and evaluation* strategy proactively, to facilitate feedback is key. *Learning and evaluation* enables understanding of what has worked and not worked in the past for public health emergency management; this in turn enables improved planning, recovery and response for the future. Learning longitudinally gives perspective on the system over time, rather than for just one incident.

*“I think we have to look back not just on the last emergency and how well we managed that. What’s our track-record in the last five years and the last ten years? And is there a kind of systemic failure, a weak point in the system still?” (Site F)*

Linkages for evaluation across the PHEP framework are important. Information systems are needed to support data collection; measures to evaluate *practice and experience*; and processes and *resources* are needed to support real-time “course correction” to promote adaptability and flexibility of PHEP systems.

*“And what I find really interesting is that, I think there’s more and more recognition around how important that phase is, and yet even though, in my perspective, we’ve moved into doing something like debriefing and even doing it together in partnership, it still isn’t necessarily translating itself into the next iteration of the planning. We don’t actually necessarily change or act on those things that we see repeatedly.” (Site A)*

Despite participants’ awareness of the importance of *learning and evaluation*, they discussed gaps in how well this element is implemented in practice. The themes of *learning and evaluation* and *practice and experience* are linked. Learning opens opportunities for positive emergence or innovation, and evaluation can enable documenting of innovations implemented during a response to inform system preparedness and revise practice. Strategies to solicit feedback or share lessons learned are important for evaluation of an event or a training exercise. Evaluation serves to document and can assist in understanding the unpredictable or disproportionate impacts or behaviours from an event that relate to non-linearity of the system, and contribute to learning for better preparedness for the next event. Sharing knowledge across systems and across jurisdictions can promote change in a broader context.

A high-level synthesis and description of the essential elements is provided in Table 3.

**Table 3:** Summary of the essential elements of public health emergency preparedness

Element	Description
Governance and leadership	Vertically and horizontally integrated structures, partnerships and accountabilities to support coordinated and interoperable system functioning Defines roles, promotes clarity and enables flexibility across system
Planning process	Dynamic process anchored in the development of relationships and clear responsibilities Supports linkages across readiness priorities and activities
Collaborative networks	Effective partnerships share skillsets and support trust in a networked system Enables access to expertise for a range of hazards and impacts
Community engagement	Inclusivity supporting proactive understanding of community priorities and values Enables consideration of community risks, assets and values, and facilitates transparency and trust
Risk analysis	Process to understand risks for the community, access and analyze information Facilitates informed planning and decision-making
Surveillance and monitoring	Robust surveillance and information processes to connect the system, key stakeholders and the community Facilitates awareness in advance and analysis of impacts of public health actions to guide response
Practice and experience	Exercises, simulations and/or practice to promote agency and create feedback Enables co-evolution and informs potential areas for adjustment
Resources	Scalable and sufficient infrastructure promote adaptive capacity and support decision-making Ensures capability for mobilizing resources linked to plans and establishing priorities for the allocation of limited resources
Workforce capacity	Well-trained and knowledgeable people constitute crucial social infrastructure for the system Supports business continuity, inter-operability and requires reciprocity
Communication	Understandable information for awareness and potential actions Enables feedback and reach to diverse audiences when supported by sufficient capacity
Learning and evaluation	Assessments key to recovery and building back better and more likely to be successful if timely and prioritized Fosters change and improvement for better preparedness and response

### Ethics and values: Core principles guiding PHEP policy and practice

The described 11 themes represent an integration of the essential elements of PHEP with relevant ethical values and processes discussed by participants. This integration of ethics with the elements was validated through knowledge user input and ethics and values were confirmed as informing the core principles at the heart of a PHEP framework.

To summarize ethical considerations which emerged as part of the elements, the following values and processes are seen as integrated within. Values discussed important to PHEP included: equity, trust, public protection, reciprocity, duty to care, stewardship and solidarity. In addition to the values which emerged, approaches to ethical decision-making and actions for PHEP included processes such as: inclusiveness, accountability, transparency, responsiveness and reasonableness. Further details on our analysis around ethics and values will be presented in a separate paper.

## Discussion

In this study, we developed an empirically-derived and theoretically-informed framework for PHEP. The framework identifies 11 essential elements of a resilient public health system and how the elements interact as a complex adaptive system. With an upstream orientation, the framework pertains to all aspects of emergency management - encompassing readiness, response and recovery - and promotion of adaptive capacity to support resilience among local/regional public health agencies. This framework addresses an important gap by contributing to the evidence base for PHEP. While overlap exists between the essential elements identified and some existing US frameworks for PHEP and emergency management [24, 56], our rigorous approach empirically defines a framework for a non-US context, supporting US approaches and expanding a conceptualization of PHEP with relevance to other countries with similar health systems to Canada.

**Fig. 1:**
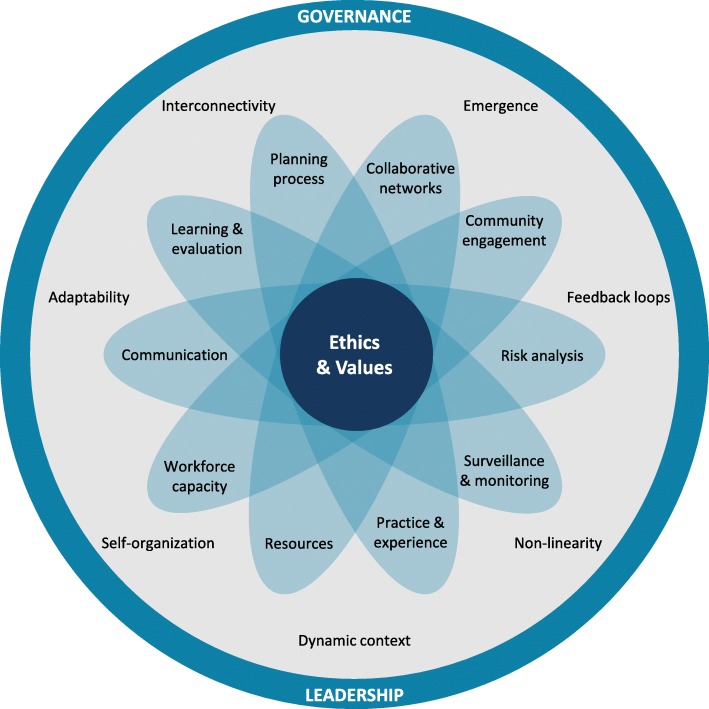
Resilience Framework for Public Health Emergency Preparedness

In developing a visual concept to represent the framework, the essential elements for PHEP found in this study, and the complexity surrounding them, were used as a starting point. Application with practice and policy relevance was an important objective; therefore, we ensured the framework resonated with knowledge users as we proceeded through different stages of the development. Figure 1 was developed iteratively and collaboratively with the research team and knowledge users through our iKT process.

### Figure 1: Resilience framework for public health emergency preparedness

Moving away from technocratic visuals, the figure is influenced by an organic image, aligning with social resilience concepts [32]. The figure reflects the interconnectedness of the elements, overlapping at the centre as a symbolic connection in the core of the framework. The elements are depicted as part of the whole, while emphasizing the cross-cutting element of *governance and leadership* encircling the stand-alone elements. The representation of *governance and leadership* surrounding the tenets of complexity theory and the 10 other elements highlights the crucial importance of *governance and leadership* as a means to facilitate and manage the dynamic, complex adaptive system of PHEP.

This framework for local/regional public health agencies addresses a knowledge gap in frameworks for this level, and also aligns with global guidelines for emergency preparedness and disaster risk reduction. The Sendai Framework for DRR (2015–2030) aims to reduce disaster risk and losses in lives, livelihoods and health [27]. Health protection and reduction of morbidity and mortality from disasters are situated within the inter-sectoral approach of Sendai [57]. Our framework supports all priorities, in particular *Priority 4: Enhancing disaster preparedness for effective response and to “Build Back Better” in recovery, rehabilitation and reconstruction*. Preparedness is described as enabling effective response and recovery; building back better is the result of resilience in the system. By using this framework to enhance preparedness, local/public health agencies can contribute to advancing progress toward the Sendai targets and the global imperative to reduce disaster risk and impact. Specific to public health, the WHO defines emergency preparedness as: “…the knowledge and capacities and organizational systems developed by governments, response and recovery organizations, communities and individuals to effectively anticipate, respond to, and recover from the impacts of likely, imminent, emerging, or current emergencies” [28]. Our resilience framework for PHEP aligns with this inclusive definition of preparedness and conceptualizes an actionable definition of preparedness that is dynamic rather than static, consistent with complexity theory.

The principles of ethics and values are conceptualized as the core of the framework, underpinning all elements and the complexity. This placement reflects the central importance of ethical principles in public health practice. A scoping review conducted by members of this team identified that ethical considerations in PHEP is a knowledge gap [15]. In emergency planning, operational frameworks have traditionally been separate from ethical frameworks [28, 58, 59]. This represents a challenge when urgent operational activities and decisions in stressful situations do not implicitly take ethics into account. The values and processes found in this study resonate with other published work on public health ethics [34, 59]; however, the integration of ethics as part of this framework and recognizing ethical values and processes at its core is a novel contribution. One salient example of how ethics and values are integrated with PHEP relates to workforce capacity. The capacity of a workforce is about having knowledgeable and skilled staff as a starting point, but there is much more to capacity building. ‘Duty to care’ and the need for organizational reciprocity and transparency in supporting staff are essential for workforce capacity and represent important aspects of ethical practice [55]. Through this element and others we identify that the human and social aspects of the framework elements enhance resilience in the system. Resilience of staff, collaboration, community engagement, leadership and the ability to learn are social dimensions that are essential for PHEP practice and this framework incorporates yet moves beyond technical competencies and physical infrastructure to emphasize how attention to social infrastructure can promote resilience in the system.

This study has limitations which are important to consider. While this framework has an upstream orientation aimed at readiness for public health agencies throughout all phases of an emergency, it is important to acknowledge that longer term impacts of disasters may not have been fully captured [8]. Linkages of public health agency practice as it overlaps with mental health care and post-disaster community recovery can be explored to consider how the elements relate to long-term population health impacts of disasters. Related to study design and to the resource-intensive nature of qualitative data collection in the field and feasibility implications, we conducted six focus groups in four provinces, rather than holding focus groups in all 10 provinces and three territories in Canada. While limited in representation, the transferability of the findings to other settings is enhanced by our purposive selection of sites across diverse geographic areas of Canada and their experience with emergency events. Further, our choice of method resulted in other advantages important for iKT and building resilience. The SIM facilitates collaboration and networking [46]; it served to bring an opportunity for each site to build its PHEP capacity through developing relationships and common ground [60].

We recognize a limitation which acknowledges Canada’s context related to its history of colonization of Indigenous peoples, their culture and lands. While representatives from First Nations health organizations were included through purposive sampling, we did not host focus groups in Indigenous communities. The research team acknowledges the disproportionate impact that emergencies may have on Indigenous communities due to different risk profiles. We recognize that future collaboration with Indigenous communities and health organizations is needed to ensure Indigenous voices are included in public health emergency planning. Given the complex governance implications of Indigenous health and emergency services, future work is needed to validate the developed framework in other settings and communities.

Any exploration of PHEP needs to consider complexity and look at various elements and interactions within a system. Complexity is inherent in health systems and is a useful lens for preparedness. The essential elements for PHEP for local/regional public health agencies are integrated within the system and the networked, interconnected and dynamic nature is reflected in the element descriptions. These descriptions inform health system activities, change and potentially support improvement by identifying actionable concepts for the field. This work thus integrates the current state of science in incorporating relevant theory to inform framework development. The framework identifies essential and potentially actionable elements relevant to change at the local/regional public health agency level.

A challenge emerges in moving from a definition for upstream activities to support resilience to measurement of a state of preparedness. Measurement and reporting of preparedness should provide support for ensuring preparedness but the typical ‘new public management’ use of measurement in health policy with a focus on benchmarking, accountabilities, and other aspects of performance management may present challenges to the use of the framework itself [61]. The framework stresses the interconnectedness of measures, an aspect of measurement and reporting that can be difficult to capture in tools like scorecards and report cards. Likewise, the use of this framework for evaluating preparedness and response to emergencies and disasters may be challenging as it does not support typical approaches to public health intervention evaluation and may require new approaches that stress concept mapping and a more sophisticated articulation of interconnectedness [44].

However, the pressure for translation of this framework or similar frameworks into measurement frameworks will likely increase. Emergencies are increasing in frequency, although may still be rare for a given jurisdiction and the culture of preparedness discussed as part of *governance and leadership* may vary in terms of establishment across public health agencies. Emergencies create public concern and often become political. Public attention on risks and impacts of emergencies can lead to significant investment in emergency management, as was seen in the last few decades in North America. In the absence of an emergency to generate political and public attention, challenges remain in organizational accountability to politicians, decision-makers and the public on the state of an agency’s preparedness, and to justify investment in preparedness. Although this study addresses a definition of readiness relevant to local/regional public health agencies in Canada and for other relevant health systems, careful consideration of how it can link to different approaches to measurement and management of the concepts represented by the framework elements may be useful to enhance practice, guide improvement and support accountability. Our future work will address these challenges.

## Conclusions

In summary, we present a conceptual framework of the essential elements for a resilient PHEP system, aimed at identifying upstream actions to promote readiness for disasters and emergencies. Our analysis describes the complexity of the system yet moves beyond description to using tenets of complexity to define a framework focused on resilience, for practice and policy action. This applied public health framework for local/regional public health agencies forms an evidentiary basis for PHEP and DRR which will be further augmented by developing key indicators.

## Additional files

Additional file 1: Current study in relation to overarching aim to advance performance measurement for public health emergency preparedness. (DOCX 27 kb)

Additional file 2: Methodology and links across phases 1 and 2 for the study Advancing performance measurement for public health emergency preparedness in Canada. (DOCX 71 kb)

Additional file 3: Consolidated criteria for reporting qualitative research (COREQ). (PDF 436 kb)

Additional file 4: Flowchart of focus groups implemented using Structured Interview Matrix (SIM) technique. (DOCX 45 kb)

Additional file 5: Coding grid applied for content analysis. (DOCX 41 kb)

## Abbreviations

DRR: Disaster risk reduction
PHEP: Public health emergency preparedness
US: United States
WHO: World Health Organization

## References

[1] Government of Alberta. Update 6: Wood Buffalo wildfire recovery (July 27 at 11 am). https://www.alberta.ca/release.cfm?xID=4292755C48F66-F699-FE35-6208390C990D8594 (2017) Accessed 11 July 2018.

[2] KPMG LLP. 2016 Wood Buffalo Wildfire, Post-Incident Assessment Report. Final Report; 2017. https://www.alberta.ca/assets/documents/Wildfire-KPMG-Report.pdf. Accessed 11 July 2018.

[3] Population, public and Aboriginal health strategic clinical network, Alberta Health Services. Overview of the strategic clinical network for population health and Aboriginal health. Alberta Health Services ; 2016. https://www.albertahealthservices.ca/scns/Page13061.aspx. Accessed 11 July 2018.

[4] MNP LLP (2016). Review and analysis of the government of Alberta's response to and recovery from 2013 floods. Government of Alberta.

[5] Moghal Z, Peddle S. At the front lines of flood: How prepared are Ontario communities. Waterloo, ON: Partners for Action; 2016. https://uwaterloo.ca/partners-for-action/sites/ca.partners-for-action/files/uploads/files/p4a_front_lines_of_the_flood_04jul16.pdf. Accessed 11 July 2018.

[6] Abbott G, Chapman M (2013). Addressing the new normal: 21st century disaster management in British Columbia. A report and findings of the BC flood and wildfire review: an independent review examining the 2017 flood and wildfire seasons.

[7] Généreux M, Petit G, Maltais D, Roy M, Simard R, Boivin S (2014). The public health response during and after the Lac-Mégantic train derailment tragedy: a case study. Disaster Health.

[8] Généreux Mélissa, Petit Geneviève, Roy Mathieu, Maltais Danielle, O’Sullivan Tracey (2018). The “Lac-Mégantic tragedy” seen through the lens of the EnRiCH Community Resilience Framework for High-Risk Populations. Canadian Journal of Public Health.

[9] Costich JF, Scutchfield FD (2004). Public health preparedness and response capacity inventory validity study. J Public Health Manag Pract.

[10] British Columbia Ministry of Health (2013). Promote, protect, prevent: our health begins here: BC's guiding framework for public health.

[11] McCabe OL, Barnett DJ, Taylor HG, Links JM (2010). Ready, willing, and able: a framework for improving the public health emergency preparedness system. Disaster med public health prep.

[12] Nelson C, Lurie N, Wasserman J, Zakowski S (2007). Conceptualizing and defining public health emergency preparedness. Am J Public Health.

[13] Nelson C, Lurie N, Wasserman J (2007). Assessing public health emergency preparedness: concepts, tools, and challenges. Annu Rev Public Health.

[14] Smith K, Jarris P, Inglesby T, Hatchett R, Kellermann A (2013). Public health preparedness research. J Public Health Manag Pract.

[15] Khan Y, Fazli G, Henry B, de Villa E, Tsamis C, Grant M (2015). The evidence base of primary research in public health emergency preparedness: a scoping review and stakeholder consultation. BMC Public Health.

[16] Centers for Disease Control and Prevention. Public health preparedness capabilities: National standards for state and local planning. Centers for Disease Control and Prevention. 2011. https://www.cdc.gov/phpr/readiness/capabilities.htm . Accessed 11 July 2018.

[17] Gibson PJ, Theadore F, Jellison JB (2012). The common ground preparedness framework: a comprehensive description of public health emergency preparedness. Am J Public Health.

[18] National Health Security Preparedness Index. Strengthening national health security and preparedness helps build a culture of health. National Health Security Preparedness Index. 2018. https://nhspi.org/ (2018). Accessed 27 Jun 2018.

[19] Stoto M (2013). Measuring and assessing public health emergency preparedness. J Public Health Manag Pract.

[20] Stoto MA, Nelson C, Savoia E, Ljungqvist I, Ciotti M (2017). A public health preparedness logic model: assessing preparedness for cross-border threats in the European region. Health Secur.

[21] National Health Security Preparedness Index. Methodology for the 2018 Release. National Health Security Preparedness Index. 2018. https://nhspi.org/wp-content/uploads/2018/04/Index_Methodology_2018.pdf . Accessed 27 Jun 2018.

[22] Potter M, Houck O, Miner K, Shoaf K (2012). National Health Security Preparedness Index: opportunity and challenges. White paper. Association of the Schools of Public Health.

[23] Public Health Agency of Canada (PHAC). About the agency: Who we are and what we do. http://www.phac-aspc.gc.ca/about_apropos/index-eng.php (2015). Accessed 20 Feb 2015.

[24] Stoto M, Nelson C, and the LAMPS investigators. Measuring and assessing public health emergency preparedness: a methodological primer. 2012. https://cdn1.sph.harvard.edu/wp-content/uploads/sites/1609/2015/04/MeasurementWhitePaper.pdf. Accessed 27 Jun 2018.

[25] European Centre for Disease Prevention and Control (2017). Public health emergency preparedness - core competencies for EU member states.

[26] Global Health Observatory (GHO) Data. Geneva: World Health Organization; 2016. http://www.who.int/gho/publications/world_health_statistics/2016/en/. Accessed 11 July 2018.

[27] United Nations Office for Disaster Risk Reduction (UNISDR) (2015). Sendai framework for disaster risk reduction 2015–2030.

[28] World Health Organization (2016). A strategic framework for emergency preparedness.

[29] Kieny MP (2014). World Health Organization media centre commentary: Ebola and health systems: now is the time for change.

[30] Centers for Disease Control and Prevention (2017). 2014–2016 Ebola outbreak distribution in West Africa.

[31] La Porte T. Organizational strategies for complex system esilience, reliability, and adaptation. In: Auerswald P, Branscomb L, La Porte T, Michel-Kerjan E, editors. Seeds of disaster, roots of response: how private action can reduce public vulnerability. First ed. Cambridge: Cambridge University Press; 2006. p. 135–53.

[32] O'Sullivan TL, Kuziemsky CE, Toal-Sullivan D, Corneil W (2013). Unraveling the complexities of disaster management: a framework for critical social infrastructure to promote population health and resilience. Soc Sci Med.

[33] Abramson DM, Grattan LM, Mayer B, Colten CE, Arosemena FA, Bedimo-Rung A (2015). The resilience activation framework: a conceptual model of how access to social resources promotes adaptation and rapid recovery in post-disaster settings. J Behav Health Serv Res.

[34] Singer P, Benatar S, Bernstein M, Daar A, Dickens B, MacRae S (2003). Ethics and SARS: lessons from Toronto. BMJ.

[35] Berstein M, Hawryluck L (2003). Challenging beliefs and ethical concepts: the collateral damage of SARS. Crit Care.

[36] Upshur REG, VanDenKerkhof EG, Goel V (2001). Meaning and measurement: an inclusive model of evidence in health care. J Eval Clin Pract.

[37] Gershon RR, Magda LA, Qureshi KA, Riley HE, Scanlon E, Carney MT (2010). Factors associated with the ability and willingness of essential workers to report to duty during a pandemic. J Occup Environ Med.

[38] Litaker D, Tomolo A, Liberatore V, Stange K, Aron D (2006). Using complexity theory to build interventions that improve health care delivery in primary care. J Gen Intern Med.

[39] Manson S (2001). Simplifying complexity: a review of complexity theory. Geoforum.

[40] Etkin D (2015). Disaster theory: an interdisciplinary approach to concepts and causes.

[41] Cilliers P (1998). Complexity and postmodernism: understanding complex systems.

[42] Cilliers P (2010). Preiser R editors. Complexity, difference and identity: an ethical perspective.

[43] Greenhalgh T, Papoutsi C. Studying complexity in health services research: desperately seeking an overdue paradigm shift. BMC Medicine. 2018;16:95. 10.1186/s12916-018-1089-4 PMC6009054 29921272

[44] Rutter H, Savona N, Glonti K, Bibby J, Cummins S, Finegood DT (2017). The need for a complex systems model of evidence for public health. Lancet.

[45] Canadian Institutes of Health Research (2015). Funding decisions database. Project title: advancing performance measurement for public health emergency preparedness in Canada.

[46] O’Sullivan T, Corneil W, Kuziemsky C, Toal-Sullivan D (2014). Use of the structured interview matrix to enhance community resilience through collaboration and inclusive engagement. Syst Res Behav Sci.

[47] O'Sullivan TL, Corneil WSIM (2013). Structured interview matrix (video).

[48] Canadian Institutes of Health Research (2012). Guide to knowledge translation planning at CIHR: integrated and end-of-grant approaches.

[49] Statistics Canada. Standard Geographical Classification (SGC). Statistics Canada. 2012.http://www12.statcan.gc.ca/census-recensement/2011/ref/dict/geo044-eng.cfm . Accessed 11 July 2018.

[50] Statistics Canada. Data and definitions. Statistics Canada. 2013. http://www.statcan.gc.ca/pub/21-006-x/2008008/section/s2-eng.htm. Accessed 11 July 2018.

[51] Statistics Canada. Health regions and peer groups. Statistics Canada. 2015. https://www150.statcan.gc.ca/n1/pub/82-402-x/2015002/hrpg-rsgh-eng.htm. Accessed 11 July 2018.

[52] Morgan DL, Gibbons LM (2008). Snowball Sampling. The Sage encyclopedia of qualitative research methods thousand oaks.

[53] Quinn PM (2015). Qualitative research and evaluation methods.

[54] Hsieh HF, Shannon SE (2005). Three approaches to qualitative content analysis. Qual Health Res.

[55] Upshur REG, Faith K, Gibson JL, Thompson AK, Tracy CS, Wilson K (2005). Stand on guard for thee: ethical considerations in preparedness planning for pandemic influenza. A report of the University of Toronto Joint Centre for bioethics, pandemic influenza working Group.

[56] The Department of Homeland Security Federal Emergency Management Agency. National response framework. 2016;Third edition https://www.fema.gov/media-library-data/1466014682982-9bcf8245ba4c60c120aa915abe74e15d/National_Response_Framework3rd.pdf. Accessed 19 Oct 2018.

[57] Maini R, Clarke L, Blanchard K, Murray V (2017). The Sendai framework for disaster risk reduction and its indicators—where does health fit in?. Int J Disaster Risk Sci.

[58] Emergency Management Policy Directorate. An Emergency Management Framework for Canada Second Edition. 2018. https://www.publicsafety.gc.ca/cnt/rsrcs/pblctns/mrgnc-mngmnt-frmwrk/mrgnc-mngmnt-frmwrk-eng.pdf. Accessed 11 July 2018.

[59] Thompson AK, Faith K, Gibson JL, Upshur REG (2006). Pandemic influenza preparedness: an ethical framework to guide decision-making. BMC Med Ethics.

[60] Kuziemsky CE, O'Sullivan TL (2015). A model for common ground development to support collaborative health communities. Soc Sci Med.

[61] Brignall S, Modell S (2000). An institutional perspective on performance measurement and management in the ‘new public sector’. Manag Account Res.

