# Supplementary material for: Public health emergency preparedness: a framework to promote resilience
Source: BMC Public Health. 2018 Dec 5;18:1344. doi: 10.1186/s12889-018-6250-7 (PMC6280369; doi:10.1186/s12889-018-6250-7)
Supplement: Supplementary file 1 — Current study in relation to overarching aim to advance performance measurement for public health emergency preparedness. (DOCX 27 kb) [file 12889_2018_6250_MOESM1_ESM.docx]

**Current study in relation to overarching aim to advance performance measurement for public health emergency preparedness**

* Current study phases 1 and 2
