# Supplementary material for: Public health emergency preparedness: a framework to promote resilience
Source: BMC Public Health. 2018 Dec 5;18:1344. doi: 10.1186/s12889-018-6250-7 (PMC6280369; doi:10.1186/s12889-018-6250-7)
Supplement: Supplementary file 2 — Methodology and links across phases 1 and 2 for the study Advancing performance measurement for public health emergency preparedness in Canada. (DOCX 71 kb) [file 12889_2018_6250_MOESM2_ESM.docx]

**QUAL data collection: SIM**

**QUAL**

**results**

**Develop framework**

**QUAL**

**data analysis**

**Phase I: Framework development**

**Indicator extraction from literature**

**Thematic analysis**

**Interpretation framework + literature**

**Develop survey instrument**

**Phase II: Indicator development**

**QUAN**

**data analysis**

**QUAN**

**results**

**QUAN**

**data collection: Delphi**

**Interpretation QUAN + qual**

**Interpretation QUAN + qual**

**Validate QUAN results with qual results**

**qual results**

**qual**

**data analysis**

**qual data collection: Delphi**

**Final set of PHEP indicators**

**Phase II: Indicator validation**
