# Supplementary material for: Public health emergency preparedness: a framework to promote resilience
Source: BMC Public Health. 2018 Dec 5;18:1344. doi: 10.1186/s12889-018-6250-7 (PMC6280369; doi:10.1186/s12889-018-6250-7)
Supplement: Supplementary file 4 — Flowchart of focus groups implemented using Structured Interview Matrix (SIM) technique. (DOCX 45 kb) [file 12889_2018_6250_MOESM4_ESM.docx]

**Data analysis approach**

Field note data used to develop coding grid

Transcripts and field note data analyzed using coding grid to identify emergent themes in a consensus-based approach with the research team; complexity applied as a lens in analysis

**SIM session wrap-up and review of themes**

Duration: 5 minutes

**SIM data collection 3: Large group discussion**

Data source: field notes, transcribed audio-recordings

Duration: 40 minutes

**SIM data collection 2: Small group discussion**

Data source: field notes, transcribed audio-recordings

Duration: 40 minutes

**SIM data collection 1: One-on-one interviews**

Data source: field notes

Duration: 45 minutes

**Overview of session**

Overview of project, objectives and relevant definitions

Duration: 15 minutes
