# Supplementary material for: Public health emergency preparedness: a framework to promote resilience
Source: BMC Public Health. 2018 Dec 5;18:1344. doi: 10.1186/s12889-018-6250-7 (PMC6280369; doi:10.1186/s12889-018-6250-7)
Supplement: Supplementary file 5 — Coding grid applied for content analysis. (DOCX 41 kb) [file 12889_2018_6250_MOESM5_ESM.docx]

| Adaptability | The ability of an organisation or group to change their practice to suit different operational contexts |
| --- | --- |
| Animals | The influence of victim’s responsibility for animals |
| Assumptions | Something that is accepted as true or as certain to happen, without proof |
| Awareness | Knowledge or perception of a situation or fact |
| Collaboration | People or organizations working together to prepare for or respond to a Public Health Emergency (PHE) |
| Communication | The ability to share information |
| Culture | Ways of being, interacting, beliefs, practices, ethos particular to a group or organisation |
| Decision-making | The action or process of making decisions in order to prepare or respond to a PHE |
| Dilemma | Making a difficult choice between two or more different possibilities |
| Equity | The unequal impact of disasters on different groups |
| Ethical Framework | Framework to guide decisions during a PHE based on pre-established values of health care system |
| Evaluation | The assessment of PHE activities for their effectiveness |
| Events | A PHE that has been used to illustrate participant’s answer |
| First Nations | A term used to describe the indigenous peoples of Canada |
| Governance | The development of Plans, strategies, policies, procedures, protocols, guidelines and their enactment |
| Incident Management System (IMS) | An organizing tool to coordinate emergency management activities throughout the Emergency Management Cycle |
| Jargon/Acronyms | Words, terms, expressions or abbreviations that are used by a particular profession or group |
| Knowledge & Skills | Activities that will increase the knowledge and understanding of staff to enable them to respond to an incident |
| Leadership | Leading a group or organization |
| Monitoring | The observation and checking of something |
| Needs | The gap between current conditions and desired condition or wants |
| Plan | The development of procedures to respond to an PHE |
| Politics | The influence (positive or negative) on health from non-health entities |
| Population | Concerning those who are not employed to respond to PHE |
| Practice | The application and performance of activities so as to acquire or maintain proficiency |
| Psychosocial | The effect of a disaster on an individual’s mental wellbeing |
| Recovery | The process of returning to situation that is the same or better than that prior to the PHE |
| Research | Activities that lead to better understanding of PHE |
| Resources | The equipment, supplies and personnel required to prepare for & respond to PHE |
| Response | The response to a Public Health Emergency |
| Risk | The evaluation of risk that a hazard will have on the health of a population |
| Roles & Responsibilities | Responsibilities, tasks or duties that a person or group is expected to complete as a function of their job |
| Rural | Challenges and opportunities in PHEP that are particular to a rural setting |
| SIM Evaluation | Participants discuss the merits and demerits of their experience if the SIM focus group |
| Systems | The organization of human resources, institutions, and physical resources that deliver services to meet the needs of populations |
| Timeliness | Done or occurring at a favourable or useful time; opportune |
| Trust |  |
| Upstream | Activities aimed at preventing an incident from becoming ‘non-routine’ |
| Values | The attribution of worth by individuals and communities |
